# Supplementary material for: Risk factors for severe disease in patients admitted with COVID-19 to a hospital in London, England: a retrospective cohort study
Source: Epidemiol Infect. 2020 Oct 13;148:e251. doi: 10.1017/S0950268820002472 (PMC7591271; doi:10.1017/S0950268820002472)
Supplement: Supplementary file 1 [file S0950268820002472sup001.docx]

**Supplementary materials**

**Supplementary Table 1: Admission blood tests and observations**

| Admission Characteristics  (no. of patients with data available) | Reference range | Percentage below reference range | Percentage above reference range | Study population median (IQR) |
| --- | --- | --- | --- | --- |
| Haemoglobin g/L (980) | 115-155 | 22.8 | 85.0 | 131 (116-143) |
| Platelets x10^9^/L (977) | 150-400 | 13.8 | 60.0 | 223 (175-288) |
| Neutrophils x10^9^/L (980) | 2.0-7.5 | 3.6 | 26.4 | 5.4 (3.8-7.8) |
| Lymphocytes x10^9^/L (980) | 1.5-4.0 | 76.1 | 1.5 | 1 (0.7-1.4) |
| Alkaline phosphatase IU/L (920) | 30-130 | 0.3 | 11.6 | 76 (59-102) |
| Alanine aminotransferase, IU/L (910) | 10-35 | 2.0 | 43.9 | 31 (20-52) |
| Bilirubin, mmol/L (917) | 0-21 | 0.0 | 4.3 | 8 (6-11) |
| Albumin, g/L (921) | 35-50 | 26.3 | 0.2 | 38 (34-40) |
| Sodium, mmol/L (987) | 133-146 | 19.5 | 58.0 | 137 (133-140) |
| Potassium, mmol/L (942) | 3.5-5.3 | 6.6 | 5.3 | 4.3 (3.9-4.6) |
| Urea, mmol/L (977) | 2.5-7.8 | 3.4 | 40.7 | 6.5 (4.4-11.2) |
| Creatinine, mmol/L (975) | - | - | - | 92 (73-154) |
| C-Reactive Protein, mg/L (975) | 0.0-5.0 | 0.0 | 96.2 | 94 (40-154) |
| Lactate, mmol/L (932) | 0.5-2.2 | 11 | 17.1 | 1.3 (0.9-1.9) |
| Pulmonary infiltrates on chest radiograph No. (%) (961) | - | - | - | 721 (75.0) |
| Heart rate (968) | 51-90 | 0.2 | 58.5 | 95 (82-110) |
| Systolic Blood Pressure (968) | 120-140 | 27.4 | 36.2 | 132 (117-148) |
| Respiratory rate, Median (IQR) (967) | 12-20 | 0.0 | 76.2 | 26 (21-32) |
| Temperature ^o^C | 36-37.9 | 3.7 | 37.8 | 37.5 (36.8-38.4) |
| Hypoxia^(*)^ No. (%) (970) | - | - | - | 534 (55.0) |
| Glasgow Coma Scale <15 No. (%) (853) | - | - | - | 122 (14.3) |

(*) Hypoxia was defined as oxygen saturations of <94% on air or the need for supplemental oxygen to maintain saturations ≥94%. Exceptions were made for two patients with established COPD who were known to retain carbon dioxide and had pre-COVID-19 saturations <94%.

Values are presented as median (interquartile range) unless otherwise specified

**Supplementary Table 2: secondary outcome – time to death or invasive mechanical ventilation with complete univariable and multivariable analysis**

| **Variable** | **Risk of death or need for IMV** | | | |
| --- | --- | --- | --- | --- |
|  | **Univariable analysis** | | **Multivariable analysis (n=754)** | |
|  | **Hazard ratio**  **(95% CI)** | **p value** | **Adjusted hazard ratio**  **(95% CI)** | **p value** |
| **Demographics** | | | | |
| Age ^(**)^ | 1.21 (1.14, 1.29) | **<0.001** | 1.13 (1.04, 1.24) | **0.005** |
| Male Sex (vs. female sex) | 1.23 (1.00, 1.51) | 0.05 |  |  |
| Ethnicity |  |  |  |  |
| White (reference) | 1 | 0.73 |  |  |
| Black | 1.07 (0.78, 1.48) |  |  |  |
| Asian | 1.15 (0.90, 1.46) |  |  |  |
| Other | 1.08 (0.72, 1.63) |  |  |  |
| **Comorbidities** | | | | |
| Diabetes (vs. none) | 1.21 (1.00, 1.47) | 0.05 |  |  |
| Hypertension (vs. none) | 1.40 (1.15, 1.69) | **<0.001** |  |  |
| Heart failure (vs. none) | 1.24 (0.92, 1.66) | 0.15 |  |  |
| Ischaemic heart disease (vs. none) | 1.22 (0.95, 1.57) | 0.12 |  |  |
| Active malignancy (vs. none) | 1.10 (0.72, 1.68) | 0.66 |  |  |
| Respiratory disease (vs. none) | 1.14 (0.91, 1.43) | 0.27 |  |  |
| **Medication usage** | | | | |
| ACEi / ARB (vs. none) | 1.13 (0.92, 1.40) | 0.24 |  |  |
| Metformin (vs. none) | 0.99 (0.78, 1.25) | 0.92 |  |  |
| Immunosuppression (vs. none) | 1.43 (0.88, 2.32) | 0.15 |  |  |
| **Admission investigations** | | | | |
| Haemoglobin ^(**)^ | 0.98 (0.93, 1.02) | 0.34 |  |  |
| Platelets ^(****)^ | 0.85 (0.77, 0.95) | **0.003** | 0.82 (0.72, 0.93) | **0.002** |
| Neutrophils ^(*)^ | 1.34 (1.22, 1.49) | **<0.001** |  |  |
| Lymphocytes ^(+)^ | Linear term 0.67 (0.48, 0.93) | **0.005** |  |  |
|  | Squared term 1.98 (1.24, 3.15) |  |  |  |
|  |  |  |  |  |
| Sodium ^(**)^ | Linear term 0.91 (0.79, 1.05) | **<0.001** | 0.81 (0.69, 0.96) | **0.01** |
|  | Squared term 1.13 (1.07, 1.92) |  |  |  |
| Potassium | 1.07 (0.90, 1.26) | **0.02** |  |  |
|  | 1.11 (1.01, 1.22) |  |  |  |
| Urea | 3.46 (2.50, 4.77) | **<0.001** | 1.82 (1.17, 2.82) | **0.008** |
| Creatinine ^(+)^ | 4.32 (2.48, 7.52) | **<0.001** |  |  |
|  | 0.33 (0.12, 0.94) ^(+)^ |  |  |  |
| C-Reactive Protein ^(***)^ | 1.32 (1.23, 1.42) | **<0.001** | 1.15 (1.08, 1.21) | **<0.001** |
|  |  |  |  |  |
| Alanine aminotransferase ^(***)^ | 0.97 (0.96, 0.99) |  |  |  |
| Alkaline phosphatase ^(+)^ | 1.67 (1.06, 2.62) | **0.03** |  |  |
| Bilirubin ^(+)^ | 1.64 (1.10, 2.46) | **0.02** |  |  |
| Albumin ^(*)^ | 0.66 (0.59, 0.75) | **<0.001** | 0.80 (0.71, 0.90) | **<0.001** |
|  | 0.91 (0.85, 0.98) |  |  |  |
| Lactate | 3.29 (2.30, 4.71) | **<0.001** | 2.88 (1.87, 4.44) | **<0.001** |
|  |  |  |  |  |
| Pulmonary Infiltrates on Chest Radiograph (vs. none) | 1.74 (1.36, 2.23) | **<0.001** | 1.90 (1.39, 2.57) | **<0.001** |
| **Admission bedside observations** | | | | |
| Heart rate ^(**)^ | 1.06 (1.01, 1.11) | **0.03** |  |  |
|  |  |  |  |  |
| Systolic BP | 0.99 (0.95, 1.03) | 0.64 |  |  |
|  |  |  |  |  |
| Respiratory rate ^(**)^ | 1.53 (1.38, 1.71) | **<0.001** | 1.34 (1.16, 1.53) | **<0.001** |
| Hypoxia (vs. none) | 1.87 (1.53, 2.29) | **<0.001** | 1.41 (1.09, 1.81) | **0.009** |
| Temperature | 0.98 (0.89, 1.07) | 0.60 |  |  |
| Glasgow Coma Score <15 (vs. 15) | 1.85 (1.44, 2.38) | **<0.001** | 1.70 (1.28, 2.25) | **<0.001** |

(*) Hazard ratios given for a 5-unit increase in variable

(**) Hazard ratios given for a 10-unit increase in variable

(***) Hazard ratios given for a 50-unit increase in variable

(****) Hazard ratios given for a 100-unit increase in variable

(+) Variable analysed on the log scale (base 10)

Variables found to be significant in univariable analysis but did not retain significance in multivariable analysis were excluded from the final model

ACEi/ARB: angiotensin converting enzyme inhibitor/angiotensin II receptor blockers. CI: confidence interval

IMV: invasive mechanical ventilation.

**Supplementary Table 3: secondary outcome – time to death, invasive mechanical ventilation or continuous positive airway pressure with complete univariable and multivariable analysis**

| **Variable** | **Risk of death, need for IMV or CPAP** | | | |
| --- | --- | --- | --- | --- |
|  | **Univariable analysis** | | **Multivariable analysis (n=754)** | |
|  | **Hazard ratio**  **(95% CI)** | **p value** | **Adjusted hazard ratio**  **(95% CI)** | **p value** |
| **Demographics** | | | | |
| Age ^(**)^ | 1.16 (1.10, 1.23) | **<0.001** | 1.10 (1.01, 1.20) | **0.02** |
| Male Sex (vs. female sex) | 1.27 (1.05, 1.55) | **0.02** |  |  |
| Ethnicity |  |  |  |  |
| White (reference) | 1 | 0.49 |  |  |
| Black | 1.18 (0.93, 1.49) |  |  |  |
| Asian | 1.15 (0.84, 1.56) |  |  |  |
| Other | 1.26 (0.85, 1.83) |  |  |  |
| **Comorbidities** | | | | |
| Diabetes (vs. none) | 1.28 (1.07, 1.54) | **0.009** |  |  |
| Hypertension (vs. none) | 1.37 (1.14, 1.65) | **0.001** |  |  |
| Heart failure (vs. none) | 1.09 (0.81, 1.45) | 0.58 |  |  |
| Ischaemic heart disease (vs. none) | 1.16 (0.91, 1.49) | 0.23 |  |  |
| Active malignancy (vs. none) | 1.02 (0.68, 1.54) | 0.92 |  |  |
| Respiratory disease (vs. none) | 1.05 (0.84, 1.31) | 0.66 |  |  |
| **Medication usage** | | | | |
| ACE / ARB (vs. none) | 1.19 (0.97, 1.45) | 0.10 |  |  |
| Metformin (vs. none) | 1.07 (0.86, 1.33) | 0.57 |  |  |
| Immunosuppression (vs. none) | 1.28 (0.79, 2.09) | 0.31 |  |  |
| **Admission investigations** | | | | |
| Haemoglobin ^(**)^ | 0.99 (0.94, 1.03) | 0.61 |  |  |
| Platelets ^(****)^ | 0.88 (0.80, 0.97) | **0.01** | 0.83 (0.74, 0.93) | **0.002** |
| Neutrophils ^(*)^ | 1.34 (1.21, 1.48) | **<0.001** |  |  |
| Lymphocytes ^(+)^ | Linear term 0.71 (0.52, 0.98) | **0.01** |  |  |
|  | Squared term 1.87 (1.19, 2.94) |  |  |  |
| Sodium ^(**)^ | Linear term 0.89 (0.78, 1.02) | **0.007** | 0.79 (0.67, 0.92) | **0.003** |
|  | Squared term 1.12 (1.06, 1.18) |  |  |  |
| Potassium | Linear term 1.07 (0.90, 1.25) | **0.02** |  |  |
|  | Squared term 1.12 (1.03, 1.21) |  |  |  |
| Urea | 3.03 (2.25, 4.09) | **<0.001** | 1.88 (1.23, 2.88) | **0.004** |
|  |  |  |  |  |
| Creatinine ^(+)^ | Linear term 4.32 (2.48, 7.52) | **<0.001** |  |  |
|  | Squared term 0.33 (0.12, 0.94) |  |  |  |
| C-Reactive Protein ^(***)^ | Linear term 1.33 (1.24, 1.42) | **<0.001** | Linear term 1.22 (1.08, 1.22) | **<0.001** |
|  |  |  | Squared term 0.98 (0.96, 1.00) |  |
| Alanine aminotransferase ^(***)^ | Squared term 0.97 (0.95, 0.99) |  |  |  |
| Alkaline phosphatase | 1.39 (0.89, 2.16) | 0.15 |  |  |
| Bilirubin ^(+)^ | 1.62 (1.11, 2.38) | **0.01** |  |  |
| Albumin ^(*)^ | Linear term 0.70 (0.62, 0.78) | **<0.001** | 0.86 (0.77, 0.96) | **0.008** |
|  | Squared term 0.93 (0.88, 0.99) |  |  |  |
| Lactate | Linear term 1.96 (1.16, 3.30) | **<0.001** | Linear term 2.27 (1.44, 3.57) | **<0.001** |
|  | Squared term 2.15 (1.09, 4.22) |  | Squared term 2.76 (1.17, 6.49) | **<0.001** |
| Pulmonary Infiltrates on Chest Radiograph (vs. none) | 1.91 (1.50, 2.43) | **<0.001** | 1.85 (1.37, 2.50) | **<0.001** |
| **Admission bedside observations** | | | | |
| Heart rate ^(**)^ | 1.06 (1.01, 1.10) | **0.02** |  |  |
|  |  |  |  |  |
| Systolic BP | 0.99 (0.95, 1.03) | 0.65 |  |  |
| Respiratory rate ^(**)^ | 1.51 (1.36, 1.68) | **<0.001** | 1.28 (1.12, 1.46) | **<0.001** |
| Hypoxia (vs. none) | 1.93 (1.59, 2.34) | **<0.001** | 1.41 (1.11, 1.80) | **0.005** |
| Temperature | 1.02 (0.94, 1.11) | 0.64 |  |  |
| Glasgow Coma Score <15 (vs. 15) | 1.62 (1.27, 2.08) | **<0.001** | 1.52 (1.15, 2.01) | **0.003** |

(*) Hazard ratios given for a 5-unit increase in variable

(**) Hazard ratios given for a 10-unit increase in variable

(***) Hazard ratios given for a 50-unit increase in variable

(****) Hazard ratios given for a 100-unit increase in variable

(+) Variable analysed on the log scale (base 10)

Variables found to be significant in univariable analysis but did not retain significance in multivariable analysis were excluded from the final model

ACEi/ARB: angiotensin converting enzyme inhibitor/angiotensin II receptor blockers. CI: confidence interval

CPAP: continuous positive airway pressure. IMV: invasive mechanical ventilation

**Supplementary table 4: Multivariable analyses examining factors associated with time to death – sensitivity analysis excluding patients with a positive swab >5 days after admission**

| **Variable** | **Risk of death** | |
| --- | --- | --- |
|  | **Multivariable analysis (n= 711)** | |
|  | **Adjusted hazard ratio**  **(95% CI)** | **p value** |
| Age ^(**)^ | 1.52 (1.36, 1.70) | <0.001 |
| Respiratory disease (vs. none) | 1.37 (1.03, 1.83) | 0.03 |
| Immunosuppression (vs. none) | 2.18 (1.20, 3.97) | 0.01 |
| Haemoglobin ^(**)^ | 0.94 (0.88, 1.00) | 0.06 |
| Platelets ^(****)^ | 0.76 (0.65, 0.89) | 0.001 |
| Alkaline phosphatase ^(+)^ | 2.64 (1.39, 5.00) | 0.003 |
| Urea ^(+)^ | 2.59 (1.58, 4.26) | <0.001 |
| C-Reactive Protein ^(***)^ | 1.17 (1.10, 1.25) | <0.001 |
| Lactate | 2.63 (1.60, 4.34) | <0.001 |
| Pulmonary infiltrates on chest radiograph (vs. none) | 1.66 (1.19, 2.32) | <0.001 |
| Heart rate ^(**)^ | 1.06 (0.99, 1.13) | 0.10 |
| Respiratory rate ^(**)^ | 1.28 (1.07, 1.53) | 0.006 |
| Hypoxia (vs. none) | 1.46 (1.08, 1.96) | 0.01 |
| Glasgow Coma Score <15 (vs. GCS 15) | 1.90 (1.38, 2.61) | <0.001 |

(*) Hazard ratios given for a 1-unit increase in variable

(**) Hazard ratios given for a 10-unit increase in variable

(***) Hazard ratios given for a 50-unit increase in variable

(****) Hazard ratios given for a 100-unit increase in variable

(+) Variable analysed on the log scale (base 10)

**Supplementary table 5: Multivariable analyses examining factors associated with time to death or invasive mechanical ventilation – sensitivity analysis excluding patients with a positive swab >5 days after admission**

| **Variable** | **Risk of death or IMV** | |
| --- | --- | --- |
|  | **Multivariable analysis (n= 724)** | |
|  | **Adjusted hazard ratio**  **(95% CI)** | **p value** |
| Age ^(**)^ | 1.13 (1.04, 1.23) | 0.008 |
| Platelets ^(****)^ | 0.79 (0.69, 0.91) | 0.001 |
| Sodium ^(**)^ | 0.76 (0.64, 0.91) | 0.002 |
| Potassium |  |  |
| Urea ^(+)^ | 1.90 (1.20, 3.00) | 0.006 |
| C-Reactive Protein ^(***)^ | 1.17 (1.10, 1.24) | <0.001 |
| Albumin ^(*)^ | 0.80 (0.71, 0.90) | <0.001 |
| Lactate | 2.99 (1.91, 4.68) | <0.001 |
| Pulmonary infiltrates on chest radiograph (vs. none) | 1.71 (1.24, 2.36) | 0.001 |
| Respiratory rate ^(**)^ | 1.34 (1.16, 1.55) | <0.001 |
| Hypoxia (vs. none) | 1.46 (1.12, 1.90) | 0.006 |
| Glasgow Coma Score <15 (vs. GCS 15) | 1.65 (1.23, 2.21) | 0.001 |

(*) Hazard ratios given for a 1-unit increase in variable

(**) Hazard ratios given for a 10-unit increase in variable

(***) Hazard ratios given for a 50-unit increase in variable

(****) Hazard ratios given for a 100-unit increase in variable

(+) Variable analysed on the log scale (base 10)

IMV: invasive mechanical ventilation.

**Supplementary table 6: Multivariable analyses examining factors associated with time to death, IMV or CPAP – sensitivity analysis excluding patients with a positive swab >5 days after admission**

| **Variable** | **Risk of death, IMV or CPAP** | |
| --- | --- | --- |
|  | **Multivariable analysis (n=724)** | |
|  | **Adjusted hazard ratio**  **(95% CI)** | **p value** |
| Age ^(**)^ | 1.09 (1.01, 1.19) | 0.04 |
| Platelets ^(****)^ | 0.82 (0.72, 0.93) | 0.002 |
| Albumin ^(*)^ | 0.86 (0.77, 0.96) | 0.009 |
| Sodium ^(**)^ | 0.75 (0.64, 0.89) | 0.001 |
| Urea ^(+)^ | 1.95 (1.26, 3.04) | 0.003 |
| C-Reactive Protein ^(***)^ | Linear term 1.21 (1.11, 1.34) | <0.001 |
|  | Squared term 0.98 (0.96, 1.00) |  |
| Lactate | Linear term 2.22 (1.41, 3.51) | <0.001 |
|  | Squared term 2.94 (1.25, 6.93) |  |
| Pulmonary infiltrates on chest radiograph (vs. none) | 1.72 (1.26, 2.36) | 0.001 |
| Respiratory rate ^(**)^ | 1.28 (1.11, 1.47) | <0.001 |
| Hypoxia (vs. none) | 1.47 (1.14, 1.89) | 0.003 |
| Glasgow Coma Score <15 (vs. GCS 15) | 1.48 (1.11, 1.98) | 0.007 |

(*) Hazard ratios given for a 1-unit increase in variable

(**) Hazard ratios given for a 10-unit increase in variable

(***) Hazard ratios given for a 50-unit increase in variable

(****) Hazard ratios given for a 100-unit increase in variable

(+) Variable analysed on the log scale (base 10)

IMV: invasive mechanical ventilation. CPAP: continuous positive airway pressure.

**Supplementary table 7: Multivariable analyses examining factors associated with time to death – sensitivity analysis with ethnicity reclassified as “BAME vs. White ethnicity”**

| **Variable** | **Risk of death: univariable analysis** | |  |
| --- | --- | --- | --- |
|  | **Univariable analysis** | |  |
|  | **Hazard ratio**  **(95% CI)** | **p value** |  |
| **Demographics** | | | |
| BAME ethnicity (vs. White ethnicity) | 0.82 (0.64, 1.04) | 0.10 |  |
| Age ^(**)^ | 1.58 (1.46, 1.71) | **<0.001** |  |
| Male sex (vs. female sex) | 1.02 (0.82, 1.23) | 0.88 |  |
| **Comorbidities and Medication Usage** | | | |
| Diabetes (vs. none) | 1.42 (1.15, 1.75) | **0.001** |  |
| Hypertension (vs. none) | 1.64 (1.33, 2.03) | **<0.001** |  |
| Heart failure (vs. none) | 1.72 (1.27, 2.32) | **<0.001** |  |
| Ischaemic heart disease (vs. none) | 1.61 (1.24, 2.09) | **<0.001** |  |
| Active malignancy (vs. none) | 1.47 (0.97, 2.25) | 0.07 |  |
| Respiratory disease (vs. none) | 1.28 (1.00, 1.63) | 0.05 |  |
| ACEi / ARB (vs. none) | 1.25 (0.99, 1.57) | 0.06 |  |
| Metformin (vs. none) | 0.97 (0.75, 1.25) | 0.81 |  |
| Immunosuppression (vs. none) | 1.68 (1.02, 2.78) | **0.04** |  |
| **Admission investigations** | | | |
| Haemoglobin ^(**)^ | 0.91 (0.86, 0.95) | **<0.001** |  |
| Platelets ^(****)^ | 0.85 (0.75, 0.95) | **0.005** |  |
| Neutrophils ^(*)^ | 1.31 (1.17, 1.47) | **<0.001** |  |
| Lymphocytes ^(+)^ | 0.58 (0.41, 0.83) | **0.001** |  |
| Sodium ^(**)^ | 1.14 (0.97, 1.35) | **<0.001** |  |
| Potassium | 1.10 (0.92, 1.33) | **0.005** |  |
| Urea ^(+)^ | 12.3 (7.34, 20.5) | **<0.001** |  |
| Creatinine ^(+)^ | 7.11 (3.75, 13.5) | **<0.001** |  |
| C-Reactive Protein ^(***)^ | 1.19 (1.13, 1.25) | **<0.001** |  |
| Alanine aminotransferase ^(+)^ | 0.59 (0.41, 0.86) | **0.006** |  |
| Alkaline phosphatase ^(+)^ | 2.63 (1.64, 4.21) | **<0.001** |  |
| Bilirubin ^(+)^ | 1.31 (0.84, 2.04) | 0.23 |  |
| Albumin ^(*)^ | 0.67 (0.59, 0.76) | **<0.001** |  |
| Lactate |  |  |  |
| Pulmonary infiltrates on chest radiograph (vs. none) | 1.27 (0.99, 1.64) | 0.07 |  |
| **Admission bedside observations** | | | |
| Heart rate ^(**)^ | 1.01 (0.96, 1.07) | **0.03** |  |
| Systolic BP ^(**)^ | 0.99 (0.94, 1.03) | **0.01** |  |
| Respiratory rate ^(**)^ | 1.45 (1.29, 1.63) | **<0.001** |  |
| Hypoxia (vs. none) | 1.77 (1.42, 2.20) | **<0.001** |  |
| Temperature | 0.93 (0.85, 1.03) | 0.17 |  |
| Glasgow Coma Score <15 (vs. 15) | 2.57 (1.98, 3.33) | **<0.001** |  |

(**) Hazard ratios given for a 10-unit increase in variable

(***) Hazard ratios given for a 50-unit increase in variable

(****) Hazard ratios given for a 100-unit increase in variable

(+) Variable analysed on the log scale (base 10)

ACEi/ARB: angiotensin converting enzyme inhibitor/angiotensin II receptor blockers

BAME: Black, Asian and minority ethnic

**Supplementary Figure 1: Kaplan-Meier plot of time to death or invasive mechanical ventilation for all patients (reduced y-axis scale). Blue shading corresponds to 95% confidence intervals**

**Supplementary figure 2: Kaplan-Meier plot of time to death, invasive mechanical ventilation or continuous positive airway pressure (reduced y-axis scale) Blue shading corresponds to 95% confidence intervals**

STROBE Statement—checklist of items that should be included in reports of observational studies

|  | Item No | Recommendation |
| --- | --- | --- |
| **Title and abstract** | 1 | (*a*) Indicate the study’s design with a commonly used term in the title or the abstract  ***The study title identifies this as a retrospective cohort study*** |
|  |  | (*b*) Provide in the abstract an informative and balanced summary of what was done and what was found  ***The abstract conveys the basic methodology, statistical methods and objective reported outcomes.*** |
| Introduction | | |
| Background/rationale | 2 | Explain the scientific background and rationale for the investigation being reported  ***The introduction gives an overview of the need for an increased knowledge of COVID-19 in hospitalized patients and summaries what is already known.*** |
| Objectives | 3 | State specific objectives, including any prespecified hypotheses  ***The aims are clearly stated: “The aim of this large UK inpatient cohort study is to explore the association between results of admission laboratory tests and clinical observations, alongside demographic and morbidity characteristics, with clinical outcomes of patients hospitalized with COVID-19.”***  ***We had no prespecified hypotheses.*** |
| Methods | | |
| Study design | 4 | Present key elements of study design early in the paper  ***This is done in the first section of the methods.*** |
| Setting | 5 | Describe the setting, locations, and relevant dates, including periods of recruitment, exposure, follow-up, and data collection  ***This is clearly stated in the ‘Study Population’ of the Methods.*** |
| Participants | 6 | (*a*) *Cohort study*—Give the eligibility criteria, and the sources and methods of selection of participants. Describe methods of follow-up  ***The eligibility criteria are clearly stated (“all adult patients (age ≥18 years) admitted to this hospital between 12 March and 15 April 2020 who were confirmed to be positive for SARS-CoV-2”). The study followed patients until the point of discharge and did not follow them up after this point.*** |
|  |  | (*b*) *Cohort study*—For matched studies, give matching criteria and number of exposed and unexposed  ***This was not a matched study.*** |
| Variables | 7 | Clearly define all outcomes, exposures, predictors, potential confounders, and effect modifiers. Give diagnostic criteria, if applicable  ***All outcomes are clearly defined in the ‘Outcomes’ section. All variables are detailed in the ‘Variables’ section of the methods*** |
| Data sources/ measurement | 8* | For each variable of interest, give sources of data and details of methods of assessment (measurement). Describe comparability of assessment methods if there is more than one group  ***Sources of data are detailed in the ‘Variables’ section of the Methods*** |
| Bias | 9 | Describe any efforts to address potential sources of bias  ***Selection bias of cases was addressed by ensuring that all patients were identified during the study period. This is noted in the Discussion.*** |
| Study size | 10 | Explain how the study size was arrived at  ***This was determined by the number of patients who met the entry during a pre-specified period. This is stated in the ‘Study Population of the Methods.*** |
| Quantitative variables | 11 | Explain how quantitative variables were handled in the analyses. If applicable, describe which groupings were chosen and why  ***Handling of variables is described in detail in the ‘Statistical Analysis’ section of the Methods.*** |
| Statistical methods | 12 | (*a*) Describe all statistical methods, including those used to control for confounding  ***This is done in detail in the ‘Statistical Analysis’ section of the Methods. Multivariable regression analysis using a Cox proportional hazards model.*** |
|  |  | (*b*) Describe any methods used to examine subgroups and interactions  ***Subgroups were not used*** |
|  |  | (*c*) Explain how missing data were addressed  ***Due to the nature of a Cox survival model the final multivariable model was based on patients who had complete data for those shown the be significant in the preceding steps. The final n values are presented in the tables for the respective outcomes.*** |
|  |  | (*d*) *Cohort study*—If applicable, explain how loss to follow-up was addressed  ***This study only included inpatients so this was no applicable.*** |
|  |  | (*e*) Describe any sensitivity analyses  ***Sensitivities analyses were done with patients with a positive COVID-19 test >5 days after admission and with reclassification of ethnicity. Both of these analyses are included in the supplemental materials.*** |

Continued on next page

| Results | | |
| --- | --- | --- |
| Participants | 13* | (a) Report numbers of individuals at each stage of study—eg numbers potentially eligible, examined for eligibility, confirmed eligible, included in the study, completing follow-up, and analysed  ***This study included all adult patients who were admitted to Northwick Park Hospital during the study period who had positive COVID-19 nasal and pharyngeal swab. No patients were excluded who met these criteria.*** |
|  |  | (b) Give reasons for non-participation at each stage ***N/A*** |
|  |  | (c) Consider use of a flow diagram ***N/A*** |
| Descriptive data | 14* | (a) Give characteristics of study participants (eg demographic, clinical, social) and information on exposures and potential confounders  ***This is discussed in the ‘Demographic and characteristics’ section of the Results and fully described in Table 1.*** |
|  |  | (b) Indicate number of participants with missing data for each variable of interest  ***This is detailed in the respective tables.*** |
|  |  | (c) *Cohort study*—Summarise follow-up time (eg, average and total amount)  ***This is stipulated in the ‘Cohort outcomes’ section of the Results.*** |
| Outcome data | 15* | *Cohort study*—Report numbers of outcome events or summary measures over time  ***This is done through captioned Kaplan-Meier curves.*** |
|  |  |  |
|  |  |  |
| Main results | 16 | (*a*) Give unadjusted estimates and, if applicable, confounder-adjusted estimates and their precision (eg, 95% confidence interval). Make clear which confounders were adjusted for and why they were included  ***In the outcome tables all variables are presented with 95% confidence intervals. Univariable and multivariable analyses are presented and clearly labelled. Due to space constraints the univariable analyses for the secondary outcomes are presented in the supplemental materials.*** |
|  |  | (*b*) Report category boundaries when continuous variables were categorized  ***Continuous variables were not categorized.*** |
|  |  | (*c*) If relevant, consider translating estimates of relative risk into absolute risk for a meaningful time period  ***This study reported hazard ratios.*** |
| Other analyses | 17 | Report other analyses done—eg analyses of subgroups and interactions, and sensitivity analyses  ***Sensitivity analyses are reported as per 12 (e).*** |
| Discussion | | |
| Key results | 18 | Summarise key results with reference to study objectives |
| Limitations | 19 | Discuss limitations of the study, taking into account sources of potential bias or imprecision. Discuss both direction and magnitude of any potential bias |
| Interpretation | 20 | Give a cautious overall interpretation of results considering objectives, limitations, multiplicity of analyses, results from similar studies, and other relevant evidence |
| Generalisability | 21 | Discuss the generalisability (external validity) of the study results |
| Other information | | |
| Funding | 22 | Give the source of funding and the role of the funders for the present study and, if applicable, for the original study on which the present article is based  ***This study received no funding.*** |
